# MicroRNA therapy for infected wounds

**DOI:** 10.15252/emmm.201809574

**Published:** 2018-09-17

**Authors:** Paul Hiebert, Sabine Werner

**Affiliations:** ^1^ Department of Biology Institute of Molecular Health Sciences Swiss Federal Institute for Technology (ETH) Zurich Zurich Switzerland

**Keywords:** Immunology, Skin

## Abstract

A novel approach for identification of functional miRNAs in skin wounds reveals miR‐223 as a key regulator of inflammation during tissue repair. The findings suggest targeting of miR‐233 as an effective strategy to enhance neutrophil activation after bacterial infection and to improve healing of infected wounds.

Chronic wounds affect millions of people worldwide and account for a massive portion of health care costs in developed countries. Estimates suggest annual costs of over $30 billion in the United States (Nussbaum *et al*, 2018). Inflammatory cells play a crucial role in orchestrating effective wound repair, and a dysfunctional and prolonged inflammatory response is a hallmark of chronic wounds (Eming *et al*, 2014). One of the most frequent complications of chronic wounds is bacterial infection. The pursuit of effective treatment strategies for wound infections has been ongoing for centuries with some ancient therapies such as silver still used in modern medicine (Han & Ceilley, 2017). Topical antibiotics are often used prophylactically to limit wound infection; however, their efficacy in improving patient outcome is under debate (Han & Ceilley, 2017). Furthermore, the risk of emergence of antibiotic‐resistant strains remains a concern. The most common pathogen responsible for wound infection is *Staphylococcus aureus,* which can be life threatening when infecting mucosal surfaces and wound sites (Salgado‐Pabon & Schlievert, 2014). Treatment of *S. aureus* infection usually involves antibiotics such as methicillin, which might favor resistant strains emergence. Consequently, studies aiming to identify better treatment options are of utmost importance.

Normal wound healing occurs in different, overlapping phases, namely hemostasis, inflammation, new tissue formation, and remodeling. Recent work has led to the discovery of numerous molecular pathways orchestrating these phases during normal tissue repair, and pharmaceutical manipulation of these pathways lies at the core of many therapeutic strategies.

The discovery of microRNAs (miRNAs) in the 1990s led to a revolution in the way scientists think about protein regulation in the cell (Lee *et al*, 1993). miRNAs are highly conserved, non‐coding RNA molecules that average ~22 nucleotides in length. They typically recognize and bind to complementary sequences on messenger RNA (mRNA) molecules, leading to their translational repression or degradation. Effective recognition and suppression of mRNA targets require miRNAs to be presented in the context of a larger protein complex commonly referred to as the microRNA‐induced silencing complex (RISC). At the core of the RISC are Argonaute (Ago) proteins, which associate with miRNAs and function to efficiently recognize and bind the miRNA to their target sequences on mRNA molecules. While it is today well accepted that miRNAs can function as critical regulators of tissue repair (Luan *et al*, 2018), the specific miRNAs involved and the wound healing phases during which they primarily function remain unclear in many cases. In this issue of *EMBO Molecular Medicine*, de Kerckhove *et al* (2018) describe a novel approach to identify functional miRNAs that are elevated in response to skin injury and identify miRNA‐223 (miR‐223) as a potential therapeutic target able to influence acute inflammation in *S. aureus‐*infected wounds (Fig 1).

**Figure 1:**
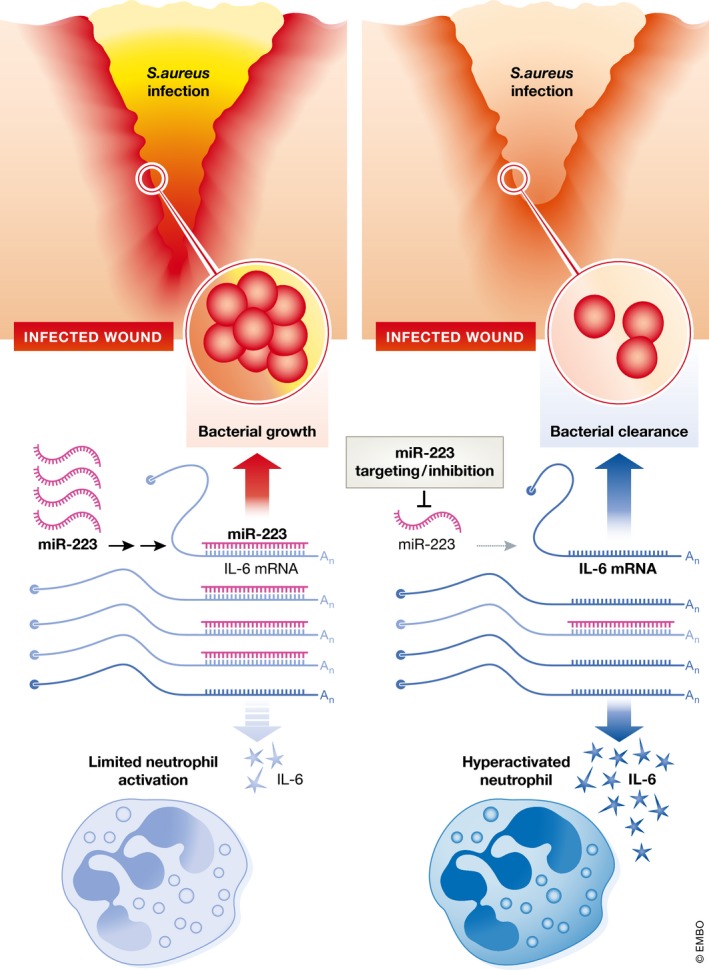
Targeting miR‐223 increases neutrophil activation in infected wounds miR‐223 expressed in neutrophils suppresses IL‐6 expression, resulting in limited neutrophil activation and enhanced bacterial growth upon *S. aureus* infection of wounds. Inhibition of miR‐223 and the resulting increase in IL‐6 production may be an effective strategy for increasing neutrophil activity during wound infection, leading to faster bacterial clearance and improved wound closure.

In their search for functional, wound ‐responsive miRNAs, the authors describe a method to specifically purify Ago‐bound miRNA complexes from mouse wounds. This resulted in the identification of hundreds of known miRNAs with a likely function in the wound healing response. Interestingly, some miRNAs showed peak expression levels 1 day following injury, indicating a role in the early phase of wound healing. The inflammatory phase begins almost immediately following injury and is of particular interest as it pertains to combating wound infection. Although several miRNAs were elevated at this stage, miR‐223 showed the highest overall expression and became the focus of the study. The strong increase in Ago‐bound miR‐223 1 day after injury suggested that it was expressed by infiltrating neutrophils and macrophages. Indeed, mutant mice lacking these cell types failed to show similar increases in miR‐223 expression following injury. Immunohistochemistry experiments using mouse wounds and human inflamed skin confirmed neutrophils and macrophages as a prominent source of miR‐223. These initial findings raise many interesting questions pertaining to the roles of other miRNAs identified during the inflammatory phase. While some of these miRNAs (e.g. miR‐142) are already known to regulate some aspects of inflammation, including *S. aureus* infection (Tanaka *et al*, 2017), the role of others remains unknown. The data also provide an important rationale for further investigating these immune‐related miRNAs, such as miR‐223, in human wounds. For example, it would be interesting to examine miR‐223 levels in immune cells of human acute vs. chronic wounds.

Targeting specific miRNAs represents a promising strategy as a means of fighting different pathologies. In their study, de Kerckhove *et al* (2018) investigate the therapeutic potential of targeting miR‐223 during wound healing using mice with a specific deletion of miR‐223 (miR‐223^Y/−^). Live imaging revealed increased recruitment of neutrophils in miR‐223^Y/−^ mice as early as 12 h post‐injury. Increased neutrophil activation and production of reactive oxygen species were also observed. This led to increased macrophage infiltration and concomitant impairment of wound healing during normal conditions, including slower re‐epithelialization and an increase in early scar tissue formation. These findings are in line with a deleterious effect of an excessive inflammatory response on wound repair and promotion of scarring (Martin & Leibovich, 2005). Interestingly, the deleterious impact on wound healing observed under pathogen‐free conditions was reversed in wounds infected with *S. aureus*, where the absence of miR‐223 also led to neutrophil recruitment/hyperactivation, but ultimately improved wound repair. Similarly, transplantation of neutrophils lacking miR‐223 into infected wounds or treatment of infected wounds with nucleic acid probes designed to bind and inhibit the activity of miR‐223 led to significantly improved healing. Overall, these results provide an important basis for targeting miR‐223 as a way of manipulating key pathways influencing the immune response during infection.

The effects of miR‐223‐specific targeting on infection in human wounds and on the resulting repair process remain to be investigated, particularly given the differences between mouse and human immune responses to infection (Salgado‐Pabon & Schlievert, 2014). These important findings therefore provide rationale for testing this hypothesis further in the clinic.

Mechanistically, the expression of miR‐223 in human neutrophils was promoted by the transcription factor C/EBPα, which is decreased upon neutrophil activation, leading to reduced miR‐223 expression. The effect of miR‐223 in murine neutrophils is driven, at least in part, by directly targeting interleukin (IL)‐6 mRNA. IL‐6 is expressed and secreted by activated neutrophils and is important for fighting infection. Importantly, human neutrophils expressed less miR‐223 upon recognizing *S. aureus*, corresponding to elevated IL‐6 secretion, an effect mimicked in non‐stimulated neutrophils with miR‐223 knock‐down.

Collectively, the authors demonstrate a key role for miR‐223 in limiting neutrophil activation. Silencing of miR‐223 effectively promoted the hyperactivation of neutrophils, leading to elevated IL‐6 secretion and improved bacterial defense. These findings highlight the therapeutic potential of targeting specific miRNAs during chronic wound infection and suggest miRNA therapy as a promising alternative to already existing strategies. Future work in this area will certainly be of great interest, as the need for better approaches for treating chronic wounds remains a major priority for health care providers.
